# ﻿A new species of *Passaloecus* Shuckard (Hymenoptera, Crabronidae) from China, with a key to Oriental species

**DOI:** 10.3897/zookeys.1181.108543

**Published:** 2023-10-10

**Authors:** Nawaz Haider Bashir, Wenbo Li, Zhuocheng Liu, Tiyuan Xia, Huanhuan Chen

**Affiliations:** 1 College of Biological Resource and Food Engineering, Qujing Normal University, Qujing 655011, China; 2 Key Laboratory for Plant Diversity and Biogeography of East Asia, Kunming Institute of Botany, Chinese Academy of Sciences, Kunming 650201, China; 3 College of Agriculture and Life Sciences, Kunming University, Kunming, Yunnan 650241, China; 4 Key Laboratory of Yunnan Province Universities of Qujing Natural History and Early Vertebrate Evolution, Qujing Normal University, Qujing 655011, China; 5 State Key Laboratory of Resource Insects, Key Laboratory of Insect-Pollinator Biology of Ministry of Agriculture and Rural Affairs, Institute of Agricultural Research, Chinese Academy of Agricultural Sciences, Beijing 100193, China

**Keywords:** Identification key, Pemphredoninae, Pemphredonini, sphecid wasp, taxonomy

## Abstract

A new species of *Passaloecus* Shuckard, *P.birugatus* Bashir & Chen, **sp. nov.**, is described and illustrated from Yunnan Province, China. The new species can be easily distinguished from known species of *Passaloecus* by its very long petiole, which is distinctly longer than wide, obscure scrobal suture, propodeum rugae and striations, body punctation, and coloration. An identification key to the Oriental species of *Passaloecus* is given.

## ﻿Introduction

The members of the genus *Passaloecus* Shuckard, 1837 (Hymenoptera, Crabronidae) are small predatory wasps. The genus belongs to the tribe Pemphredonini, subtribe Pemphredonina (Pulawski 2023). The subtribe Pemphredonina are recognized by their forewing, which has three discoidal cells and two recurrent veins (Kim and Yang 2010). Among genera of the subtribe Pemphredonina, *Passaloecus* is differs from the closely related genus *Polemistus* de Saussure in lacking long setae on the ventral gena, having the inner orbits almost parallel, and a rarely found omaulus; from other genera, *Diodontus* Curtis and *Pemphredon* Latreille, in this subtribe, *Passaloecus* differs in having the labrum roundly produced, a horizontal hypersternaulus, hind-tibia lacking a series of spines, a complete episternal sulcus, and females without a pygidial plate (Bohart and Menke 1976; Bashir et al. 2021). Females of *Passaloecus* build internal cell partitions from plant resins and construct their nests in soil, stems, soft wood, or abandoned insect nests (Antropov and Perkovsky 2009; Kim and Yang 2010).

*Passaloecus* currently comprises 45 species and five subspecies worldwide (Tsuneki 1955, 1967, 1974; Merisuo 1972, 1973; Bohart and Menke 1976; Vincent 1979; Rezaei et al. 2020; Pulawski 2023). Several studies have documented this genus from China (Ma et al. 2013, 2018; Bashir et al. 2019, 2021), and, as a result of the present work, 17 species and one subspecies of *Passaloecus* are known from China, among them four species and one subspecies from Oriental China, and five species are distributed in both Palaearctic and Oriental China. Prior studies have described five new Oriental species, *P.columnaris* (Yunnan), *P.labrinigratus* (Yunnan), *P.multituberculatus* (Zhejiang), and *P.petiolatus* (Tibet) (Ma and Li 2012), and, most recently, *P.frontirugatus* (Ningxia, Liaoning, and Zhejiang) (Bashir et al. 2021) from China.

The present paper records a new species of the genus *Passaloecus*, described and illustrated from the Oriental Region of China, and provides an illustrated identification key to the Oriental *Passaloecus*.

## ﻿Materials and methods

Specimens examined were collected from Yunnan Province, China, using Malaise traps. Specimens were observed with the help of a Nikon microscope (SMZ745). For the terminology, we follow Bohart and Menke (1976), Harris (1979), and Bashir et al. (2020). Photographic images were taken using a Leica (S8APO) stereoscopic microscope attached to a computer, processed using Adobe Photoshop 8.0, and prepared into plates using Adobe Illustrator (2019). Measurements and ratios were acquired as the maximal length with an ocular scale on a Nikon microscope (SMZ745). The description of the new species is based on the holotype, and the differences in the paratypes are given between square brackets.

The abbreviations are used in the species descriptions as follows:

**AOD** Distance from inner eye margin to antennal socket, frontal view;

**EDL** Distance between inner eye margins at base of clypeus, frontal view;

**EDU** Distance between inner eye margins at base of vertex, dorsally;

**ELL** Eye length in lateral view, maximum;

**EWF** Eye width in front view, maximum;

**EWL** Eye width lateral view, maximum;

**GWL** Gena width in lateral view, maximum;

**HLD** Head length in dorsal view, the distance from occipital margin to frons, medially;

**HLF** Head length in front view, the distance from the clypeal margin to the vertex, medially;

**HW** Head width, dorsally;

**IAD** Distance between antennal sockets, frontal view;

**LFI** Length of flagellomere I;

**LFII** Length of flagellomere II;

**LMTI** Length of metasomal tergum I, dorsally, maximum;

**OD** Ocellocular distance, distance between inner orbit and outer margin of hind ocellus, dorsally;

**OOD** Ocello-occipital distance, distance between occipital margin and posterior margin of hind ocellus, dorsally;

**PD** Postocellar distance, distance between inner margins of hind ocelli, dorsally;

**PL** Pedicel length;

**PLL** Petiole length laterally, maximum;

**PWD** Petiole width dorsally, medially;

**SL** Scape length;

**WAS** Width of antennal socket, frontal view;

**WFI** Width of flagellomere I;

**WFII** Width of flagellomere II;

**WMTI** Width of metasomal tergum I, dorsally, maximum.

## ﻿Taxonomy

### ﻿Genus Passaloecus

Taxon classificationAnimaliaHymenopteraCrabronidae

Shuckard, 1837

A4324126-E008-5CD9-B9E0-A7BC63D9FB23

#### Type species.

*Pemphredoninsignis* Vander Linden, 1829.

### ﻿Identification key to Oriental species of *Passaloecus*

Females of *P.multituberculatus* Ma & Li and *P.petiolatus* Ma & Li, and males of *P.frontirugatus* Bashir & Ma, *P.labrinigratus* Ma & Li, and *P.monilicornistaiwanus* Tsuneki remain unknown.

**Table:** 

1	Six visible gastral terga (Fig. 1); 10 flagellomeres (Fig. 2) (females)	**2**
–	Seven visible gastral terga (Fig. 3); 11 flagellomeres (Fig. 4) (males)	**10**
2	Mandible tridentate apically (Fig. 5)	***P.columnaris* Ma & Li**
–	Mandible bidentate apically (Fig. 6)	**3**
3	Petiole longer than wide (Fig. 7)	***P.birugatus* sp. nov.**
–	Petiole wider than long (Fig. 8)	**4**
4	Scutal patches present (Fig. 10; yellow circle area)	**5**
–	Scutal patches absent (Figs 11, 12)	**6**
5	Gaster between segments I and II slightly constricted; scutellum with midsize punctures (Fig. 10); notauli extending to one third of scutum; antero-lateral corner of pronotal collar moderately produced	***P.labrinigratus* Ma & Li**
–	Gaster between segments I and II distinctly constricted; scutellum with fine punctures (Fig. 11); notauli present on scutum only anteriorly; pronotal collar without antero-lateral corner	***P.bisulcatus* Bashir & Ma**
6	Free margin of clypeus truncate (Fig. 13)	**7**
–	Free margin of clypeus concave or round (Figs 14, 15)	**8**
7	Scrobal sulcus deeply grooved, weakly crenate (Fig. 16; yellow rounded rectangle area); notauli present on scutum only anteriorly; admedian line distinct; scutum without rugae posteriorly (Fig. 11)	***P.frontirugatus* Bashir & Ma**
–	Scrobal sulcus very weakly impressed, not crenate (Fig. 17; yellow rounded rectangle area); notauli reaching one third of scutum length; admedian line weakly impressed; scutum with short, longitudinal rugae posteriorly (Fig. 12; yellow rounded rectangle area)	***P.insignis* (Vander Linden)**
8	Gaster between segments I and II distinctly constricted; propodeal enclosure reticulate; admedian line distinct; labrum slightly constricted subapically; clypeal free margin rounded	***P.clypealis* Faester**
–	Gaster between segments I and II not constricted; propodeal enclosure rugose; admedian line weakly impressed; labrum distinctly constricted subapically (Fig. 14); clypeal free margin concave	**9**
9	Pronotal lobe ivory to yellowish	***P.monilicornismonilicornis* Dahlbom**
–	Pronotal lobe black	***P.monilicornistaiwanus* Tsuneki**
10	Spinose tubercles on hind margin of gastral tergum VI mesally present (Fig. 9)	**11**
–	Spinose tubercles on hind margin of gastral tergum VI mesally absent (Fig. 3)	**13**
11	Propodeal enclosure and posterior surface of propodeum reticulate; admedian line distinct; labrum triangular, not constricted subapically (Fig. 13)	***P.multituberculatus* Ma & Li**
–	Propodeal enclosure and posterior surface of propodeum rugose; admedian line weakly impressed; labrum distinctly constricted subapically (Fig. 14)	**12**
12	Gaster between segments I and II not constricted; flagellomeres II–VIII beneath with thin, raised tyloids; clypeal free margin concave (Fig. 14)	***P.monilicornis* Dahlbom**
–	Gaster between segments I and II constricted; flagellomeres IV–VIII beneath with narrow, long tyloids; clypeal free margin truncate (Fig. 13)	***P.insignis* (Vander Linden)**
13	Petiole longer than wide (Fig. 7)	**14**
–	Petiole wider than long (Fig. 8)	**15**
14	Flagellomeres III–IX beneath with tyloids; admedian line distinct	***P.birugatus* sp. nov.**
–	Flagellomeres IV–VIII beneath with tyloids; admedian line weakly impressed	***P.petiolatus* Ma & Li**
15	Mandible tridentate apically (Fig. 5); lower frons shiny	***P.columnaris* Ma & Li**
–	Mandible bidentate apically (Fig. 6); lower frons coriaceous	**16**
16	Scrobal sulcus distinct, crenate (Fig. 16; yellow rounded rectangle area); notauli distinct; sternum I without keel	***P.bisulcatus* Bashir & Ma**
–	Scrobal sulcus lacking (Fig. 18; yellow rounded rectangle area), or very weakly impressed (Fig. 17; yellow rounded rectangle area); notauli weakly impressed; sternum I with a slender, longitudinal keel medially	***P.clypealis* Faester**

### ﻿ Passaloecus birugatus

Taxon classificationAnimaliaHymenopteraCrabronidae

Bashir & Chen sp. nov.

BA2BFEA4-631C-532B-B929-6C30E3EAFC13

https://zoobank.orgE802C056-6BD1-454C-B9F9-81C1D4BA729F

Figs 19–30 , 31–32

#### Type materials.

***Holotype*: China** ♀; Yunnan, Shangri-La city, Shangri-La Alpine Garden; 27°90'N, 99°64'E; 8.VI.2020, 3269 m elev.; No. 202051101; coll. Huanhuan Chen. ***Paratypes***: 1♀, same data as for holotype, except No. 202051103; 2♀♀, same data as for holotype, except 15.VII.2021, No. 20214001, 20214002; 1♂, same data as for holotype, except No. 202051102. Specimens are deposited in the Insect Collection of Qujing Normal University, Qujing, Yunnan Province, China.

#### Diagnosis.

The new species can be easily separated from the similar species *P.frontirugatus* by the following (characters of *P.frontirugatus* in brackets): ocellar triangle and vertex behind ocelli finely and sparsely punctate (ocellar triangle and vertex behind ocelli with midsize punctures, close to each other); anterior carina of pronotal collar lacking (strong anterior carina present); notauli distinctly impressed (notauli slightly impressed); mesopleuron posteriorly without longitudinal rugae (mesopleuron posteriorly with short, sparse, longitudinal rugae); petiole distinctly longer than wide (petiole distinctly shorter than wide). The male can be distinguished from the closely related Oriental species *P.petiolatus* by a distinct interantennal tubercle; flagellomeres III–IX beneath with narrow, long tyloids; admedian line and notauli distinct; scutellum finely punctate; and patterns of propodeum rugae.

**Figures 1–9.**
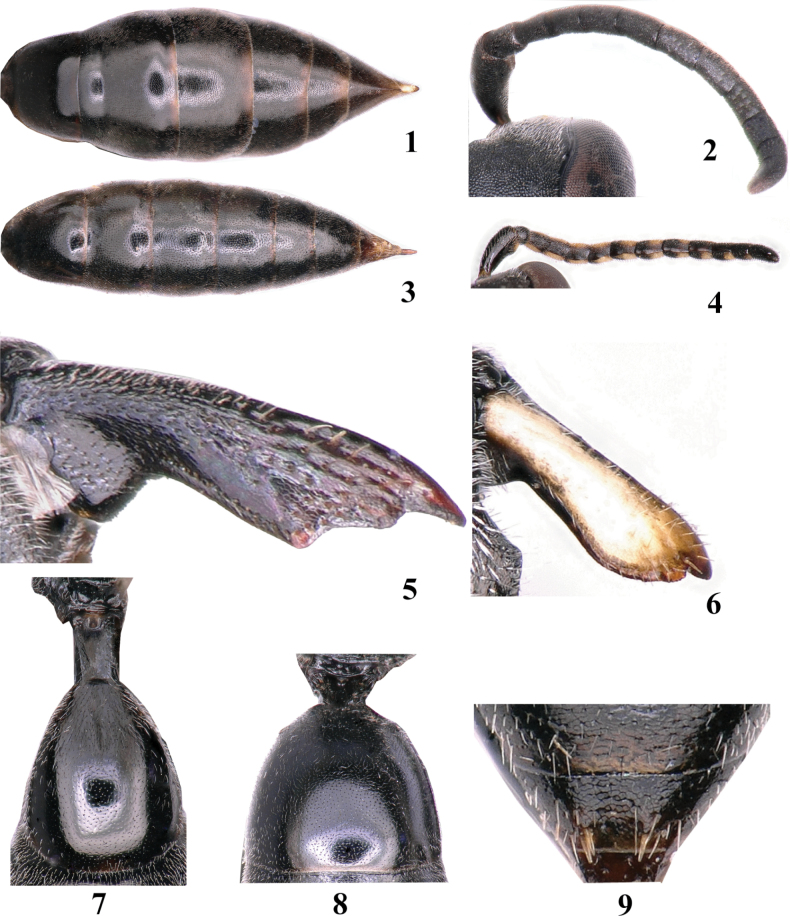
Genus *Passaloecus***1** female metasoma, dorsal view **2** female antenna **3** male metasoma, dorsal view **4** male antenna **5, 6** mandible **7, 8** petiole, dorsal view **9** gastral tergum VI, dorsal view.

**Figures 10–18.**
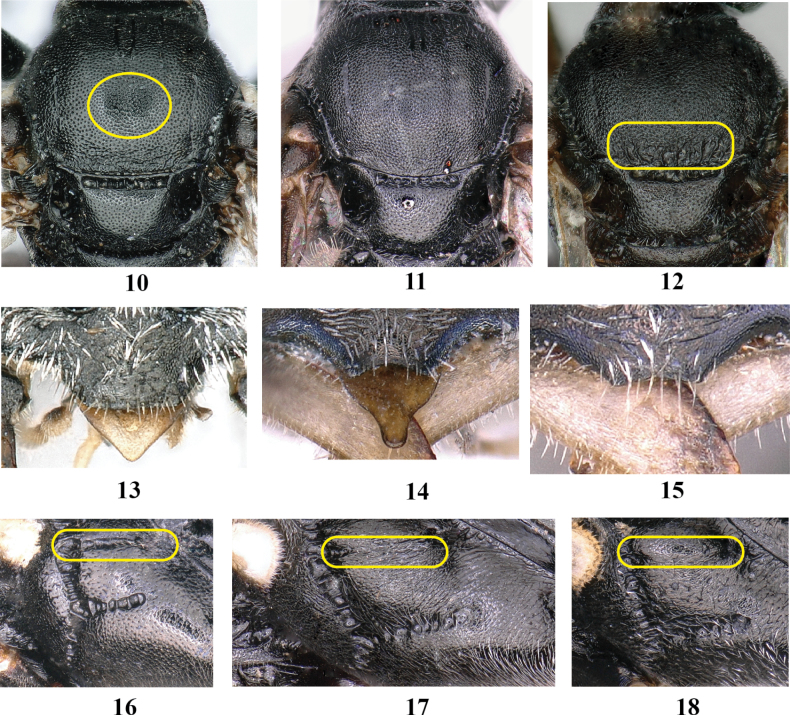
Genus *Passaloecus***10–12** thorax, dorsal view **13–15** clypeus, frontal view **16–18** mesopleuron.

#### Description.

**Female** (Figs 19–27, 31), body length 6.5 [6.2–6.6] mm.

***Colour pattern***: body black except the following: labrum, tegula and mandible apically reddish brown, remaining mandible ivory to yellowish [yellow]; palpi ivory [yellow]; scape ivory beneath, black above; pronotal lobe creamy white; forewing veins dark brown and hindwing veins light brown; tibiae and tarsi reddish brown to fulvous, remaining legs black; clypeal setae silvery (Fig. 31).

**Figures 19–30.**
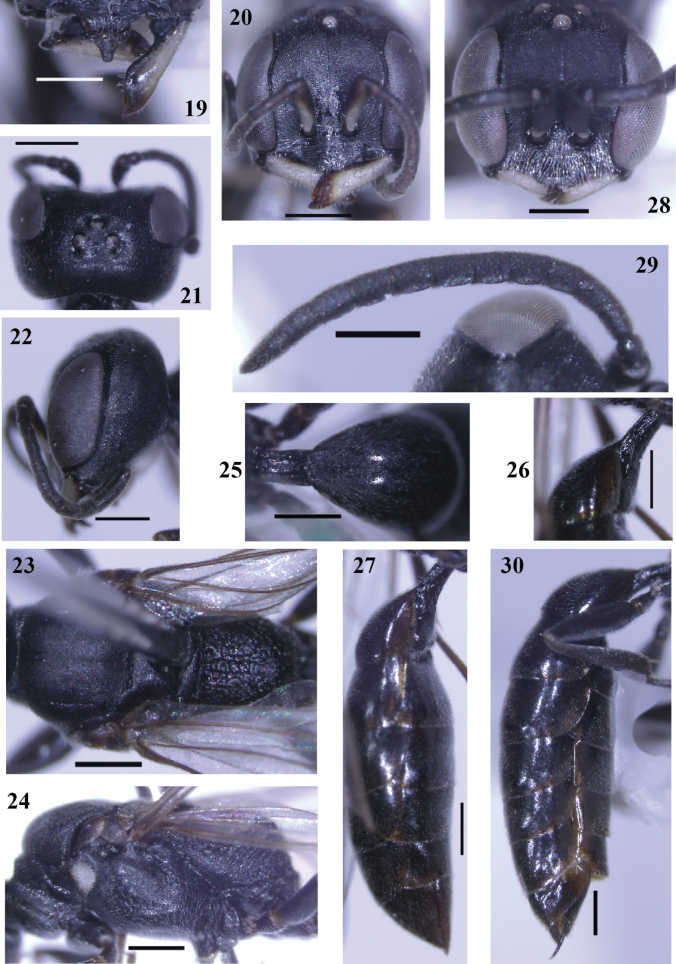
*Passaloecusbirugatus* sp. nov. **19** clypeus, frontal view **20, 28** head, frontal view **21** head, dorsal view **22** head, lateral view **23** thorax, dorsal view **24** thorax, lateral view **25** petiole, dorsal view **26** petiole, lateral view **27, 30** metasoma, lateral view **29** antenna, dorsal view (Figs 19–27 female, 28–30 male). Scale bars: 400 µm.

***Head***: mandible bidentate apically (Figs 19, 20); labrum constricted subapically and slightly wider than clypeal free margin (Fig. 19); clypeus somewhat convex medially (Fig. 20); clypeal free margin produced medially, slightly convex (Fig. 20); clypeal setae 0.2–0.3 mm long, sparse (Fig. 20); upper and median frons densely (punctures 1–2× diameters apart), finely punctate, coarsely coriaceous; inter-antennal tubercle distinct and short, frontal median area slightly impressed; lower frons coarsely coriaceous (Fig. 20); ocellar triangle finely, sparsely punctate (punctures 3–4× diameters apart), slightly coriaceous, slightly convex (Fig. 21); vertex behind ocelli with slender, sparse, transverse striations, strongly coriaceous, finely, sparsely punctate (Fig. 21); upper gena coarsely coriaceous with fine punctures 1–2× diameters apart (Fig. 22); lower gena slightly coriaceous, finely, sparsely punctate; occipital carina single, without crenulation (Fig. 21); HLF: HW: HLD = 65: 84: 50; ELL: EWL: GWL: EWF = 58: 25: 30: 17; WAS: AOD: IAD = 8: 11: 10; EDU: EDL = 52: 50; PD: OD: OOD = 11: 15: 22; SL: PL: LFI: LFII: WFI: WFII = 25: 10: 8: 9: 6: 6.

**Figures 31–32.**
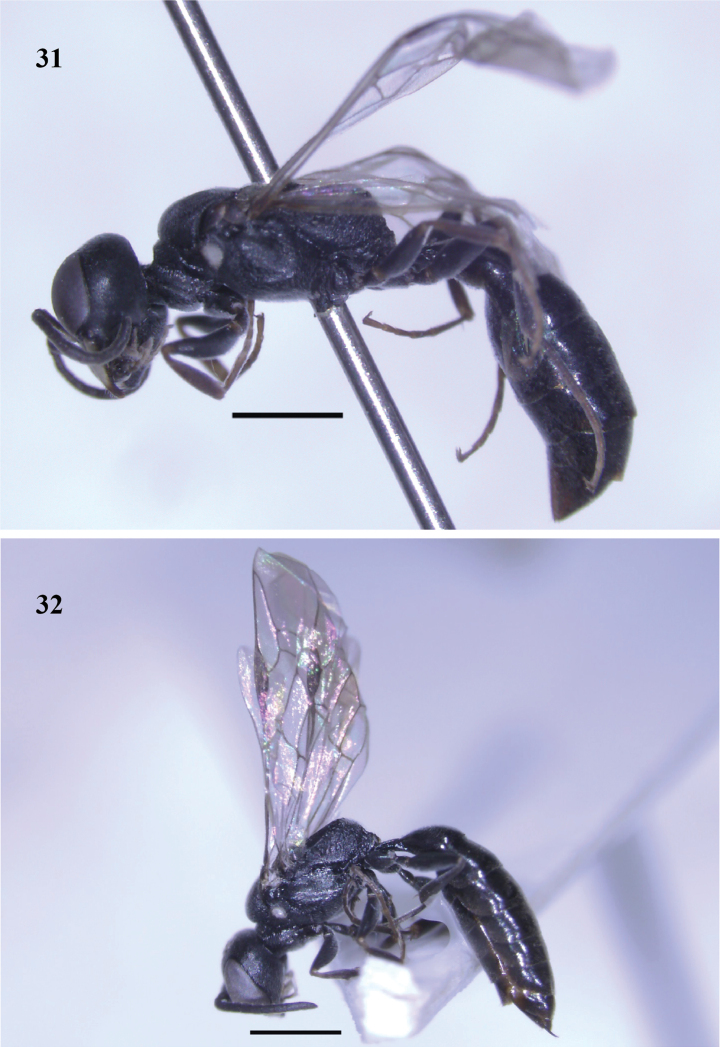
*Passaloecusbirugatus* sp. nov. **31** female, lateral view **32** male, lateral view. Scale bars: 1000 µm.

***Mesosoma***: pronotal collar anterior transverse carina lacking, antero-lateral corners slightly produced (Fig. 23), anterior slope of scutum vertical and high (Fig. 24); scutum with fine to midsize, dense punctures (punctures 0–2 diameters apart), coarsely coriaceous, scutal patches absent (Fig. 23); admedian line distinct, more than 1/3 of scutum length; notauli distinctly impressed, extending to 1/4 of scutum length, slightly shorter than admedian line; parapsidal line long (300 µm), distinct (Fig. 23); scutellum shiny, with fine, dense punctures (punctures 0–1 diameters apart); metanotum finely punctate (punctures 1–2 diameters apart); mesopleuron slightly coriaceous, with fine, sparse punctures, posteriorly without longitudinal rugae; scrobal sulcus weakly impressed, forming a thin line, as long as third hindtarsomere length (Fig. 24); omaulus absent; episternal sulcus and hypersternaulus distinctly crenate (Fig. 24); metapleuron shiny; propodeal enclosure ecarinate, not delimited laterally, with two strong, longitudinal rugae and irregular, strong, oblique transverse rugae medially (Fig. 23); posterior surface with irregular, dense, slender, transverse rugae; lateral surface with oblique, slender, dense, longitudinal striations anterodorsally, without rugae below (flat area), with sparse, weak, longitudinal rugae posteriorly (Fig. 24).

***Metasoma***: petiole longer than wide (Figs 25, 26); gaster finely, sparsely punctate, slightly coriaceous, dull (Fig. 27); sternum I in anterior half with slender, longitudinal keel (Fig. 26); sternum II slightly impressed basally; gaster not constricted between segments I and II; pygidial plate lacking; PLL: PWD: LMTI: WMTI = 25: 10: 60: 42.

**Male** (Figs 28–30, 32): same as female, except body length 5.9 mm; labrum not constricted subapically; setae on clypeus dense (Fig. 28); clypeal free margin truncate (Fig. 28); frons with midsize punctures (punctures 0–1 diameters apart); flagellomeres III–IX beneath with narrow, long tyloids, distal part of flagellomeres V–VIII curved beneath (Fig. 29); propodeal enclosure with five strong longitudinal rugae medially and laterally; sternum I without longitudinal keel medially; basal 1/5 of scape beneath ivory, remainder black (Fig. 28); HLF: HW: HLD = 57: 73: 40; ELL: EWL: GWL: EWF = 49: 24: 21: 17; WAS: AOD: IAD = 8: 7: 9; EDU: EDL = 48: 38; PD: OD: OOD = 8: 13: 16; SL: PL: LFI: LFII: WFI: WFII = 17: 8: 9: 8: 6: 6; PLL: PWD: LMTI: WMTI = 23: 10: 60: 37.

#### Distribution.

China (Yunnan).

#### Etymology.

The name *birugatus* is derived from the Latin prefix *bi*- (= two) and the Latin word *rugatus* (= rugose), with reference to the propodeal dorsal with two strong longitudinal rugae.

## Supplementary Material

XML Treatment for Passaloecus

XML Treatment for Passaloecus birugatus

## ﻿Additional information

### Author ORCIDs

Nawaz Haider Bashir https://orcid.org/0000-0001-9627-9920

Zhuocheng Liu https://orcid.org/0000-0003-1586-7945

Huanhuan Chen https://orcid.org/0000-0001-9477-1014

### Data availability

All of the data that support the findings of this study are available in the main text.
